# The promises and pitfalls of functional magnetic resonance imaging hyperscanning for social interaction research

**DOI:** 10.1111/spc3.12707

**Published:** 2022-09-12

**Authors:** Lily Tsoi, Shannon M. Burns, Emily B. Falk, Diana I. Tamir

**Affiliations:** ^1^ School of Psychology and Counseling Caldwell University Caldwell New Jersey USA; ^2^ Department of Psychological Science Pomona College Claremont California USA; ^3^ Department of Neuroscience Pomona College Claremont California USA; ^4^ Annenberg School for Communication University of Pennsylvania Philadelphia Pennsylvania USA; ^5^ Department of Psychology University of Pennsylvania Philadelphia Pennsylvania USA; ^6^ Wharton Marketing Department University of Pennsylvania Philadelphia Pennsylvania USA; ^7^ Operations, Information, and Decisions Department University of Pennsylvania Philadelphia Pennsylvania USA; ^8^ Department of Psychology Princeton University Princeton New Jersey USA; ^9^ Princeton Neuroscience Institute Princeton University Princeton New Jersey USA

**Keywords:** hyperscanning, naturalistic stimuli, neuroscience methods, social interaction

## Abstract

Social neuroscience combines tools and perspectives from social psychology and neuroscience to understand how people interact with their social world. Here we discuss a relatively new method—hyperscanning—to study real‐time, interactive social interactions using functional magnetic resonance imaging (fMRI). We highlight three contributions that fMRI hyperscanning makes to the study of the social mind: (1) Naturalism: it shifts the focus from tightly‐controlled stimuli to more naturalistic social interactions; (2) Multi‐person Dynamics: it shifts the focus from individuals as the unit of analysis to dyads and groups; and (3) Neural Resolution: fMRI hyperscanning captures high‐resolution neural patterns and dynamics across the whole brain, unlike other neuroimaging hyperscanning methods (e.g., electroencephalogram, functional near‐infrared spectroscopy). Finally, we describe the practical considerations and challenges that fMRI hyperscanning researchers must navigate. We hope researchers will harness this powerful new paradigm to address pressing questions in today's society.

## 1 INTRODUCTION

A typical day is rife with social interactions: the coffee shop barista handing you a steaming cup of coffee; a colleague chitchatting with you in the hallway; a friend exchanging rapid‐fire texts during an argument; parents across different time zones conversing with you over the phone; your significant other telling you about their hard day over dinner. Social interactions—the dynamic and interdependent experiences enacted between two or more people—are a pervasive part of daily life. It is integral for the study of human psychology to describe and understand the key components of social interaction and their consequences for our thoughts, feelings, behavior, and well‐being.

Social neuroscience promises unique insights into social interactions by examining the psychological and neural processes that give rise to social thoughts, feelings, and behaviors. It combines tools and perspectives from social psychology and neuroscience to inform theories of how people are influenced by their social environment. Although traditional approaches in social neuroscience study one person at a time or asynchronous interactions, a relatively new method called hyperscanning has emerged as a key tool for studying real‐time, interactive social phenomena. In this review, we discuss how hyperscanning with functional magnetic resonance imaging (fMRI), in particular, can help us understand the naturalistic, dyadic, and dynamic nature of social interactions beyond what typical approaches can achieve.

## 2 WHAT IS fMRI HYPERSCANNING?

Hyperscanning focuses on naturalistic social interactions in which multiple people can converse or engage in different forms of social interaction in real‐time. With hyperscanning, researchers can track dynamic interactions, like conversations, as they unfold and are co‐created by more than one brain.

Hyperscanning offers a shift from prior work in social neuroscience. In paradigms that don't involve hyperscanning, the experiment is typically scripted by the experimenter, limiting real‐time engagement; or one brain is typically scanned at a time (either someone sitting next to the scanner or outside of the scanner room), limiting measurement of dual‐brain dynamics. In contrast, hyperscanning can capture the dynamics of naturalistic social interaction by collecting neural data from multiple participants at once. Typically, each participant is scanned with a separate neuroimaging device while interacting via an audio link, video feed, or shared digital platform. Hyperscanning can use scripted elements like watching a movie together (Golland et al., 2015), semi‐scripted elements like discussing experimenter‐generated prompts (Spiegelhalder et al., 2014), or entirely unscripted elements like freely moving one's hands with meaningless gestures (Dumas et al., 2010) or having an open‐ended conversation. Together with theories from the fields of communication, psychology, and neuroscience, hyperscanning offers a way to systematically and scientifically bridge our understanding of the brain with the ways that people interact in real life. Hyperscanning with fMRI, in particular, allows researchers to leverage the benefits of magnetic resonance imaging (MRI) to provide high spatial resolution and whole‐brain coverage.

**FIGURE 1:**
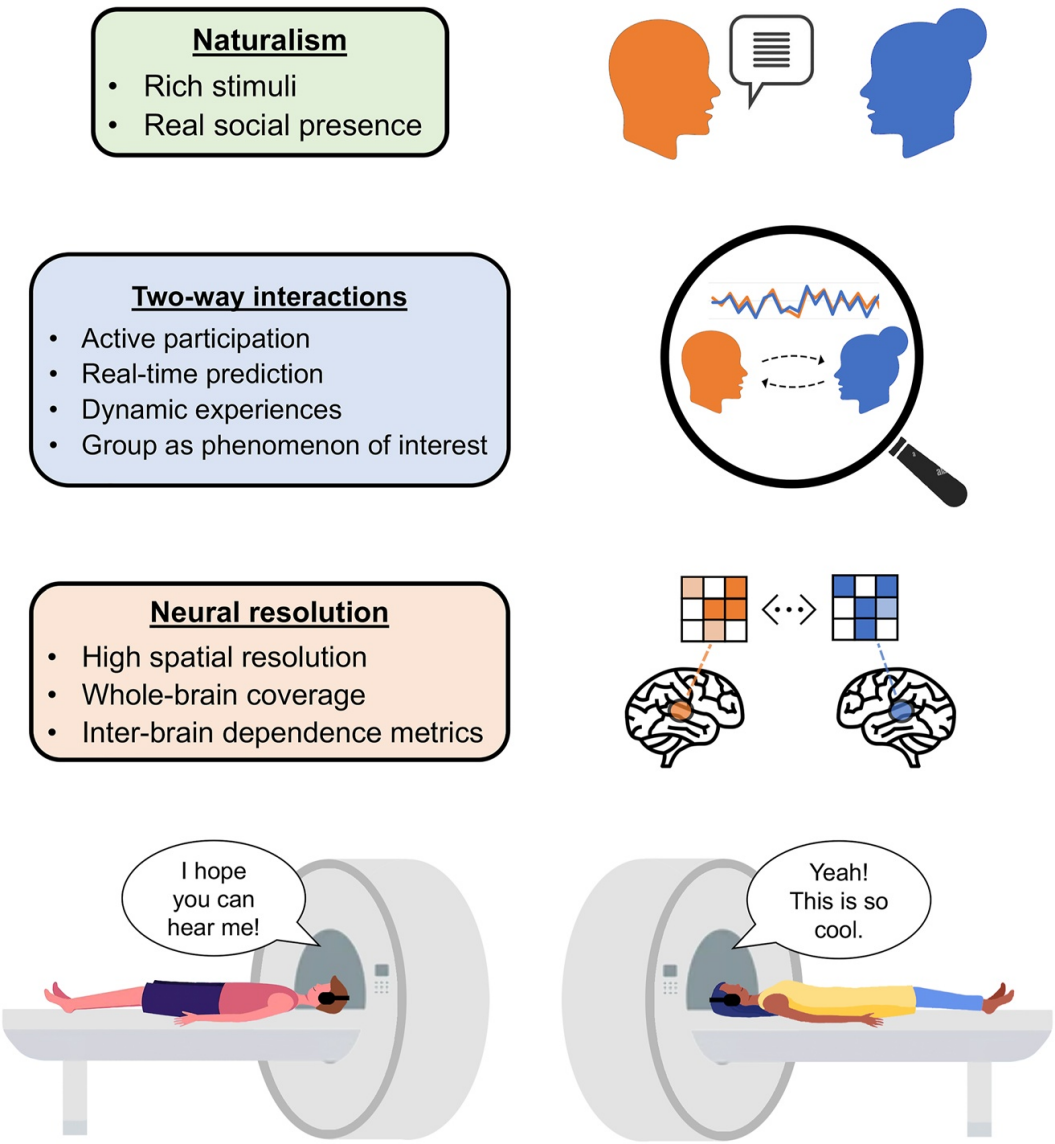
Three contributions of fMRI hyperscanning to the study of the social mind

In the current review, we highlight three contributions that fMRI hyperscanning makes to the study of the social mind (Figure 1): (1) Naturalism: it shifts the focus from relying on tightly‐controlled stimuli to depicting more dynamic social interactions; (2) Multi‐Person Dynamics: it shifts the focus from individuals as the unit of analysis to treating dyads and groups as the item of interest; and (3) Neural Resolution: fMRI hyperscanning allows researchers to consider the spatial distribution of brain activity patterns and dynamics across the whole brain instead of relying on a small number of brain regions or oscillations from coarsely‐localized sources, such as in other neuroimaging hyperscanning methods like functional near‐infrared spectroscopy (fNIRS) or electroencephalogram (EEG). We illustrate how analytic innovations enable these contributions, and the knowledge fMRI hyperscanning has given us so far. We then describe the practical considerations and challenges of fMRI hyperscanning that researchers need to navigate to use the paradigm appropriately. Finally, we conclude by describing how researchers can use fMRI hyperscanning to address open questions that are among the most pressing in our current societies.

## 3 WHY USE HYPERSCANNING

### 3.1 Shifting toward naturalism

People's cognitions (e.g., how they think, what they attend to) are influenced by their current context, past experience (e.g., upbringing), and future goals (Gilovich et al., 2016). One primary goal of social psychology and social neuroscience is to uncover and characterize the influences of context on people's thoughts and behaviors. By ‘context,’ here we refer to the situational influences that may impact people's behaviors, in particular, the presence or actions of another person. To understand humans in context, social neuroscience researchers have to *provide* context in the scanner. This necessity has led to a shift from using more constrained stimuli to more naturalistic stimuli (e.g., Hasson et al., 2004), and in particular, a shift toward including real interactions between people (Fan et al., 2021; Schilbach et al., 2013).

Consider the heartbreak you felt the moment your first romantic relationship ended. Next, imagine the different types of stimuli that one could present while you're in the MRI scanner to evoke these feelings: a picture of a heart breaking; a photo of your ex; a song that was special in your relationship; a recording of your ex breaking up with you. Each stimulus can only partially evoke the actual thoughts and feelings you had, if at all. If one were to study heartbreak, simple and constrained stimuli may not be sufficient to capture the rawness of the emotions that one may experience.

Historically, researchers avoided naturalistic stimuli for fear of being unable to interpret or isolate individual processes with naturalistic data (Nastase et al., 2020). Recent methodological innovations make it possible to have the best of both worlds. Researchers are now using more naturalistic stimuli like entire movies with multiple characters that move through time and space, with an abundance of visual and audio information hitting the senses. To make sense of this type of naturalistic data, researchers examine the temporal dynamics between people's brains. For example, researchers can measure how one person's brain responses match another's brain responses as they both sync with the naturalistic stimuli. Using this approach, researchers have discovered that engaging movies and stories reliably evoke remarkably similar brain responses in audience members (Hasson et al., 2004; Schmälzle et al., 2015; Stephens et al., 2010), especially when they come to the same high‐level interpretation of a narrative (Yeshurun et al., 2017). Naturalistic stimuli can address questions for which more tightly‐controlled stimuli are less equipped. For instance, different media or forms of communication vary in the richness of information they provide. Richer media (e.g., Zoom call vs. a text message) allows for more effective interpersonal communication, which, in turn, improves affect and connection (media richness theory; Daft & Lengel, 1986; Sheer, 2020). This is just one example of how naturalistic media in experiments can help us to understand how rich stimuli, with multiple simultaneous cues (e.g., a PSA with auditory cues such as somber music and visual cues such as people coughing and getting sick) impacts people's thoughts, feelings and behaviors (e.g., smoking tendencies).

In the same vein, studying more naturalistic social interactions—by including real interactions with other agents—can lead to greater insight into social cognition that simpler paradigms cannot capture. Consider your thoughts and actions in three different scenarios: (1) you walk past a sign that encourages you to donate to a cause; (2) you walk past the same sign, this time held by a person who doesn't look at or engage with you; (3) you walk past the same sign, this time held by a friendly person who chats with you about the cause. Your mental states (e.g., your desire to donate; your desire to portray yourself as a generous person; your thoughts about people impacted by the issue) and behaviors (e.g., your donation amount) may differ across these three scenarios. Why might this be the case?

First, the mere presence (perceived or real) of other people impacts people's thoughts (Alkire et al., 2018; Gilbert, 1998; Redcay et al., 2010; Rice & Redcay, 2016). One key aspect of naturalistic social interactions is the presence of another agent, like the person holding the sign. The *perceived* presence of another person engages greater processing than when a person is in the perceived presence of a non‐human entity like a computer. For instance, interacting with avatars that people think are controlled by humans versus a computer elicits greater activity in the reward system (Pfeiffer et al., 2014). Thinking that the outcome of a two‐player game is dependent on the interaction partner's response versus the computer's response elicits greater activity in mentalizing regions (Tsoi et al., 2016). When people make eye contact with another live person versus eye contact with a photo, the prefrontal cortex is engaged (Cavallo et al., 2015; Hirsch et al., 2017). Brain regions implicated in social and affective processes, such as the amygdala and anterior cingulate, are engaged more during real moral decisions (in which a participant's response could lead a confederate to receive painful shocks) than during hypothetical moral decisions (FeldmanHall et al., 2012).

Simply put, the brain responds differently to ‘real’ situations. Studying interactions with computers pales in comparison to studying interactions with agents perceived to be human. Studying interactions with perceived human agents may pale in comparison to studying interactions with actual human agents. Hyperscanning thus offers a unique window into naturalistic interaction by studying responses to real humans.

However, the mere presence of another person does not alone offer a wholly naturalistic social interaction. A second key aspect of naturalistic interactions is that interacting agents are interdependent (Figure 1). Namely, one person's thoughts and behaviors influence and are influenced by others' thoughts and behaviors. Interdependency is formed when people are present *and* actively engaging with one another (Lange & Balliet, 2012; Thibaut & Kelley, 1959).

### 3.2 Moving from one‐way to two‐way interactions

Cognition during real‐time interaction is fundamentally different from cognition during individual components of an interaction (Redcay & Schilbach, 2019). Imagine that the person holding the donation sign points at the sign, and you respond by looking at the sign. Engaging in joint attention, when compared to individual components of joint attention (e.g., how people respond to how a person shifts their gaze), elicits increased activity in regions implicated in mentalizing, reward processing, and attention (Caruana et al., 2015; Mundy, 2018; Oberwelland et al., 2016; Redcay et al., 2012; Schilbach et al., 2010). Suppose the sign holder also chats with you, transmitting information about the cause to you, and listening to your expressed concerns. In that case, their brain activity can predict your brain activity; this relationship is specific to the two of you and not to any two individuals (Stephens et al., 2010). In short, real interactions evoke processes beyond those evoked by individual components of the interaction, and the evoked neural dynamics are specific to the interacting interdependent agents and not to any pairing of random individuals.

**FIGURE 2:**
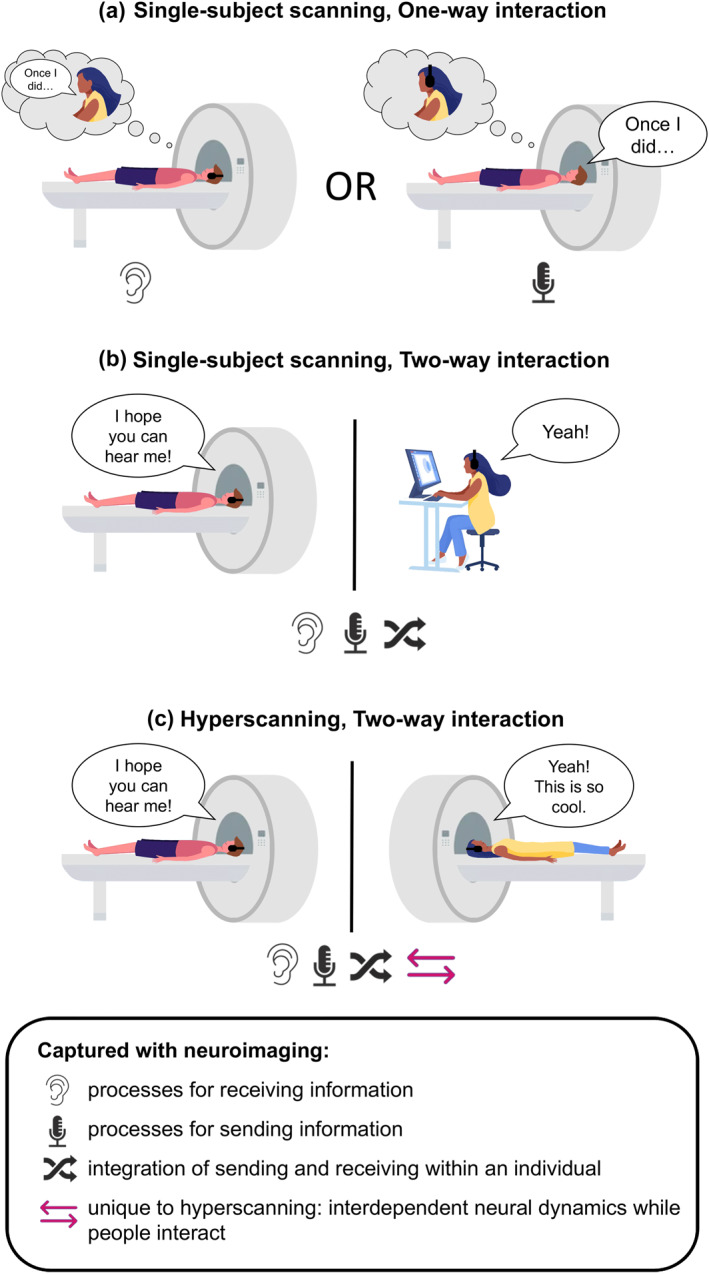
Different ways in which neuroscientists study social interactions and benefits of each way. In each subfigure, at least one person has their brain activity measured; here, we have fMRI, but it could be with other methods as well (electroencephalogram, functional near‐infrared spectroscopy) (a) Single‐subject scanning, one‐way interaction: Either the scanned individual receives input from another individual but cannot reciprocate (e.g., when listening to a story told as an audience member) or the scanned individual communicates to their partner but the partner cannot reciprocate (e.g., when telling a story to an audience). These methods capture one‐sided aspects of social interactions (b) Single‐subject scanning, two‐way interaction: the scanned individual engages in real‐time interactions with another individual who is not being scanned (they can be outside of the scanner room or inside the scanner room with the participant). (c) Hyperscanning, two‐way interaction: two or more interaction partners are scanned simultaneously while interacting with one another. For sample questions that can be uniquely addressed with hyperscanning, see Table 1

To date, most neuroscience research on social interactions does *not* have people engage in real‐time interaction (Figure 2). Instead, they focus on individual components of social interactions, akin to one‐way interaction, which provides little back‐and‐forth between agents. This type of interaction is like being an actor in a movie performing to an audience. An audience member can engage with and react to what the actor is saying and doing, but the actor cannot see or respond to that audience member in turn, and the audience cannot react with the actor's response in mind. In neuroimaging paradigms of one‐way communication, participants may experience the same naturalistic stimuli, but they do not actually interact with each other (Simony et al., 2016); or one participant may share a story with others, but the listeners cannot respond (Stephens et al., 2010). Real social interactions involve more than just these kinds of shared experiences. You likely don't feel socially bonded to every other audience member in a movie theater. What changes when communication shifts from one‐way to deeper two‐way interactions? We propose that hyperscanning allows researchers to uniquely probe the psychology of naturalistic, dyadic social interactions in at least three ways:

First, during social interactions, communicators are *active*. Communicators do not passively experience a stimulus; they listen and generate information in turns. In other words, they participate in creating the stimuli at hand. Choosing what to say depends on actively working to understand, so even listening becomes more than a passive act. This entails a more engaged and complex psychological experience than listening to communication as a third party with little ability or responsibility to influence that communication. Indeed, hyperscanning research has revealed how this interactive engagement matters: the more realistic the communication (e.g., bi‐directional vs. monologue; face‐to‐face vs. back‐to‐back), the greater the neural coupling between communicators (Kinreich et al., 2017; Liu et al., 2017). Thus, hyperscanning can help answer open questions regarding changes in neural activity (e.g., magnitude, network dynamics) during active versus passive communication.

Second, active communicators must *predict* each other's thoughts, feelings, words, and actions (Bach & Schenke, 2017; Brown & Brüne, 2012; Koster‐Hale & Saxe, 2013; Tamir & Thornton, 2018; Thornton et al., 2019). Neuroimaging offers a unique window into prediction by allowing us to measure where and how neural activity in one brain anticipates the behaviors and neural activity of another. In this way, hyperscanning can address questions probing how prediction supports successful communication. For example, during interactions between people of different statuses (e.g., between a leader and follower, teacher and student), is an influential leader someone who anticipates and calls to mind the responses of the follower, or someone who effectively gets others to align to them? Initial work into this question reveals that leaders monitor followers' responses and closely synchronize their brain activities with their followers (Jiang et al., 2015; Sänger et al., 2012, 2013; Sievers et al., 2020; but see Konvalinka et al., 2014; Zhou et al., 2016).

Third, social interactions are *dynamic*. Interaction outcomes depend on the history of what happened in the interaction, and processes within it vary over time. We can think about the relevance of time in several ways. For instance, interlocutors cumulatively update their beliefs and values. Successful production and understanding of communicative acts build on all that was said before. These processes can interact over time in ways that are not easily modeled by simply averaging a variable during the interaction or measuring a single output at the end. In social psychology, for example, a dynamical systems approach has been successful in identifying ways in which attitudes are distributed in a social group by examining the trajectory of relevant factors (e.g., nonlinearity of attitude change, geometry of the social space) over time (Vallacher & Nowak, 1997). Hyperscanning allows researchers to interrogate these dynamics by recording brain activity throughout an interaction rather than just a snapshot or final state.

**TABLE 1:** List of analyses applicable for functional magnetic resonance imaging (fMRI) hyperscanning

Analysis	Goal	Example question	Resources to learn more
Neural similarity			
Inter‐subject correlation (ISC)	Measure similarities in the temporal fluctuations of neural activity between brains in one region	Do empathic dyads show more neural alignment than less empathic dyads?	Hasson et al. (2004) and Nastase et al. (2019)
Spatial ISC	Measure similarities in neural representations (as patterns of spatial activity) between brains at one time point	Are neural representations between two people more similar during periods of high empathy?	Chen et al. (2017) and Zadbood et al. (2017)
Inter‐subject functional connectivity (ISFC)	Measure similarities in the fluctuations of neural activity in one region of one brain and other regions in a different brain	Do greater levels of empathy between two people lead to greater inter‐subject alignment across brain networks?	Simony et al. (2016)
Temporal dynamics			
Cross‐correlation analysis	Measure neural alignment offset in time, where one person's activity precedes or follows the other's	Do successful empathizers show greater neural prediction of their partner's neural activity than weak empathizers?	Stephens et al. (2010)
Granger causality analysis	Measure the direction of information flow from one brain to another	When eliciting empathy from others, do disclosers' brain activity predict their empathizer's brain activity?	Schippers et al. (2010)
Dynamic structures			
Complementary brain states	Measure how brain states coordinate across people with different roles in an interaction	Does successful empathy lead to more coordinated state changes between a discloser and empathizer?	Hasson and Frith (2016)
Graph theory measures	Measure the geometric structure of neural networks	Are between‐brain networks more tightly clustered for more empathic dyads?	Sänger et al. (2012)
Linking brain and behavior signatures			
Linking neural data with behavioral signatures	Measure how psychological variables captured in linguistic data, video data, or behavior relate to neural dynamics	Do verbal expressions of empathy increase neural alignment? Does affective mirroring increase neural alignment?	Chen et al. (2017) and Chang et al. (2021)

*Note*: Our example questions are all focused in one domain (the study of empathy), but these methods can be used to study a wide range of questions about communication and social thought.

In sum, exchanging information between people is a distinct experience from third‐party observation in terms of the active engagement, prediction, and dynamics involved. In fact, the interplay between processes of communicating participants (how these processes integrate and depend on one another) is so integral to communication that it no longer makes sense to use the most common analytic approach from psychology and neuroscience: focusing on the individual as the unit of analysis. Instead, it is more fruitful to consider the participants in a social interaction as a system with its own emergent properties.

### 3.3 The group as the unit of analysis

Placing research participants in socially interactive environments allows researchers to elicit and record naturalistic social psychological processes. Akin to how breaking a bar of steel down into its constituent atoms cannot reveal insights into how torsion forces impact the strength of the bar of steel, breaking a conversation into its constituent parts cannot capture how dynamics during a conversation impact the quality of the conversation. Instead, treating the social group (e.g., a dyad, team) as the unit of analysis might reveal insights into social interactions that we cannot glean otherwise. For example, research on romantic couples emphasizes how individual experiences (e.g., financial troubles) affect not just the individual but the dyad (Bodenmann, 1995, 1997; Lyons et al., 1998). In turn, the success of dyadic coping depends upon not only individual actions (Lazarus & Folkman, 1984), but rather similarity and congruence between partners' coping styles (Cronkite & Moos, 1984; Revenson, 1994).

**FIGURE 3:**
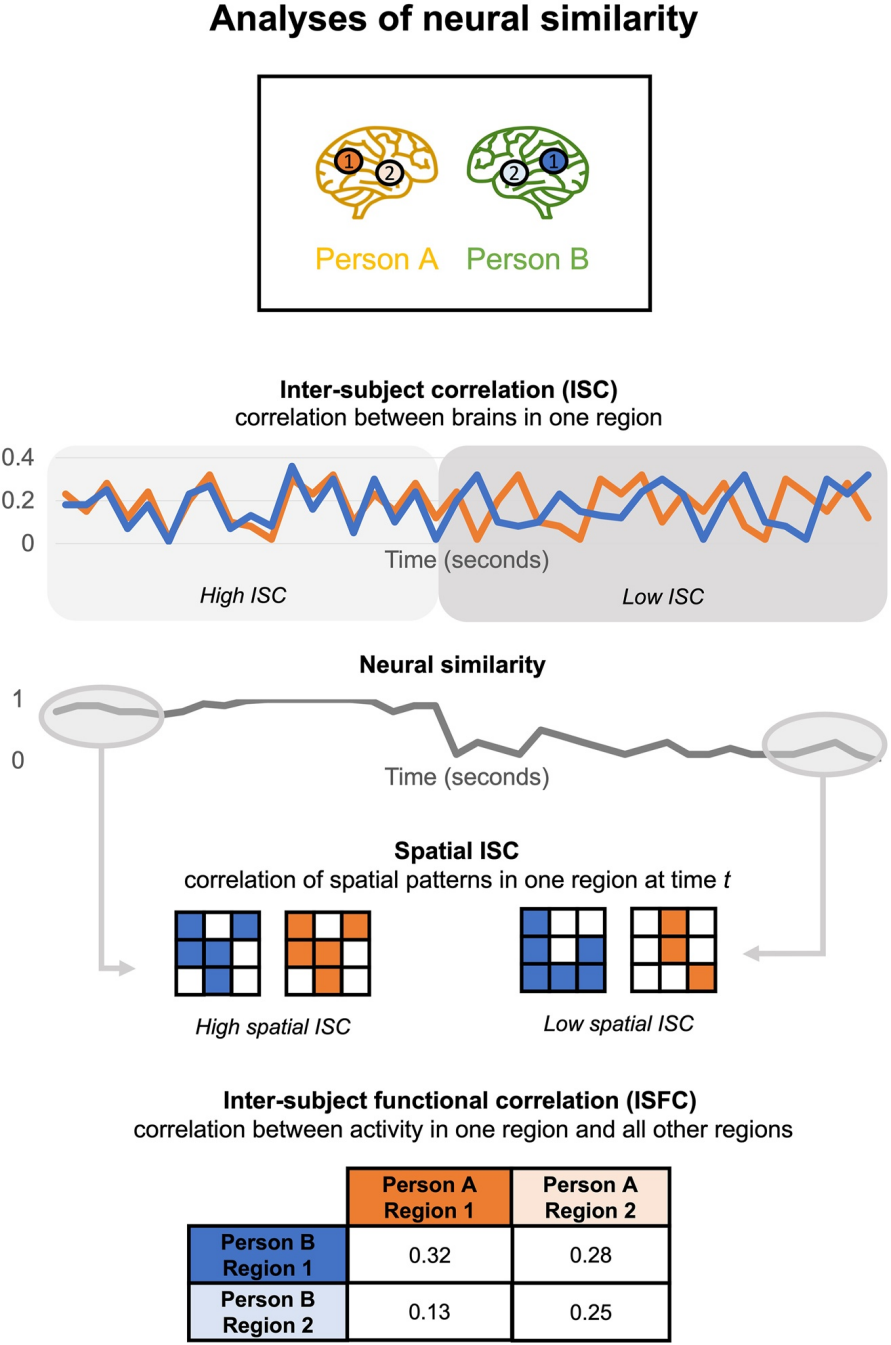
Schematic of typical analyses of neural similarity used during hyperscanning. The terms and methodological details differ for analyses of functional magnetic resonance imaging (fMRI), electroencephalogram, Magnetoencephalography, and functional near‐infrared spectroscopy hyperscanning data

Collecting brain data from multiple people simultaneously during hyperscanning allows researchers to document interdependent brain systems in an interaction. One of the simplest and most common ways of analyzing the group as a unit is to measure neural synchrony, or the congruence between partners' brain responses (Figure 3, Table 1). This neural synchrony is a marker of *mental* synchrony. Just as behavioral research reveals that people ‘on the same page’ exhibit similar language (Garrod & Pickering, 2004, 2009), body movements (Church et al., 2014; Shockley et al., 2003), and physiology (Konvalinka et al., 2011), neuroimaging research shows that people display highly synchronous brain activity when they are ‘on the same page’ about the content and interpretation of a naturalistic narrative (Stephens et al., 2010; Yeshurun et al., 2017). Behavioral, linguistic, and neural synchrony are associated with positive social outcomes, such as emotional support (Doré & Morris, 2018), interpersonal liking (Ireland et al., 2011; Putman & Street, 1984; Street et al., 1983), social cohesion (Konvalinka et al., 2011), perceptions of similarity (Valdesolo & DeSteno, 2011), prosociality (Tunçgenç & Cohen, 2018), and cooperation (Wiltermuth & Heath, 2009). Thus, prior work suggests a strong link between alignment and efficient functioning as a social group.

Hyperscanning can capture other complex relationships between people beyond synchronization (Misaki et al., 2021). For instance, in many social interactions, people do not perform the same exact actions or share the same internal states; instead, people may have complementary roles (e.g., an empathizer trying to calm an anxious person). These types of relationships cannot be captured well with synchronization. Instead, researchers can implement analyses examining complementary brain states within a dyad or group (Hasson & Frith, 2016). To measure how between‐brain networks impact social group function, researchers could use a graph theory approach (Czeszumski et al., 2020). This approach can examine how characteristics of network structures (e.g., small‐worldness: how tightly clustered a network is and how short the paths are between nodes) change during an interaction. For instance, the small‐worldness of between‐brain networks was enhanced during periods of musical coordination (Sänger et al., 2012). Finally, to measure how information flows from one person to another, researchers can examine temporal dynamics with methods such as Granger causality (Granger, 1969; Seth et al., 2015). For instance, this method can map how an observer's brain echoes the brain of a gesturer during a game of charades (Schippers et al., 2010, 2011).

Although many of these analyses can be performed on hyperscanning data from any modality (e.g., EEG, fNIRS, Magnetoencephalography (MEG), fMRI), those that capture whole‐brain dynamics can only be performed on fMRI hyperscanning data. In the next section, we elaborate on the advantages of fMRI hyperscanning for studying social interactions.

## 4 THE VALUE OF fMRI FOR HYPERSCANNING RESEARCH

Most of the hyperscanning work that has contributed to our understanding of the active, dynamic social system has used imaging modalities such as EEG and fNIRS (for reviews of studies, see Babiloni & Astolfi, 2014; Czeszumski et al., 2020; Liu et al., 2018). Both technologies are portable, making them good choices for studying research participants during natural, face‐to‐face social experiences. However, both are limited in how much information they can measure about the human brain. Both methods have lower spatial resolution than fMRI, making it challenging to map measured activity to specific brain areas (Huettel et al., 2009). For example, EEG can directly measure the electrical pulsations of neural populations from all through the head but introduces problems with identifying the specific sources (Grech et al., 2008); fNIRS can measure hemodynamic activity across the cortical surface resolved to ∼1 cm but cannot measure activity from subcortical structures (Ferrari et al., 2004).

Newer technologies like wearable magnetoencephalography with optically pumped magnometers (OPM‐MEG; Boto et al., 2018) provide an exciting addition to this area of study (Holmes et al., 2021). Wearable MEG systems are incorporated into helmets, which allow researchers to scan freely moving participants. These systems also capture high resolution temporal data, as well as higher spatial resolution than EEG. However, MEG is still less precise for deep brain structures than fMRI. We look forward to future work utilizing the new technology.

In contrast, fMRI measures whole‐brain activation, currently to ∼1 mm spatial resolution, capturing activity in both surface cortical and deeper subcortical brain regions. Psychological processes that support social thinking and naturalistic function often involve localized activity in subsurface regions (e.g., amygdala, nucleus accumbens; Adolphs, 2010; Bhanji & Delgado, 2014; Zadbood et al., 2017). A neuroimaging modality that can access and differentiate these sources of brain activity is necessary for recording the full scope of socially‐related functions.

In addition, the whole‐brain nature of fMRI also allows researchers to track mental representations via spatially‐distributed activity patterns *within* brain regions rather than extracting a single value for the average amount of activity across a region (Figure 3, Table 1). Whereas heightened activity in the temporoparietal junction (TPJ) can indicate that someone is thinking about the mental states of someone else, the specific pattern of activity within the TPJ can indicate the content of that thought (i.e., *which* mental state is being represented; Norman et al., 2006; Weaverdyck et al., 2020). This same multivariate approach can also be applied to the whole brain to decode complex mental states such as pain (Wager et al., 2013), emotion (Kragel & LaBar, 2015; Saarimäki et al., 2016), or self‐regulation (Cosme et al., 2020).

An fMRI hyperscanning approach can help identify which cognitive processes are engaged during an interaction, pinpoint periods when a cognitive process is engaged, and how interaction partners' representations or experiences relate to one another. For example, researchers can correlate interdependence metrics (e.g., the extent to which conversation partners share similar mental representations; changes in both people's mental representations following a conversation) with social outcomes (e.g., interpersonal liking, comprehension accuracy, and behavior change). These analyses can help answer broader questions, like whether interpersonal synchrony is important or how much people predict the content of others' communications. Alternatively, they can be used as dependent variables to track the social impact of interaction features (e.g., intergroup statuses between people or communication strategies).

Compared to other neuroimaging modalities, fMRI offers the best chance to capture the full scope of brain dynamics during hyperscanning. In particular, the whole‐brain dynamics uniquely captured with fMRI hyperscanning offer the potential to understand how different processes within an individual and interactions between processes across individuals impact social interactions. In this review, we include a table of published fMRI hyperscanning empirical papers thus far (Table 2).

**TABLE 2:** List of published fMRI hyperscanning studies

First author	Year	Topic	Interaction task
Montague	2002	Social exchange	Deception game: A Sender decides whether to lie about a color on the screen to a Receiver, who then guesses whether the Sender is lying
King‐Casas	2005	Economic exchange	Trust game: An Investor invests money with a Trustee, the money appreciates, and the Trustee decides how much to repay to the Investor
Tomlin	2006	Economic exchange	See King‐Casas et al. (2005)
Fliessbach	2007	Social comparison	Number estimation task: Two people estimate the number of dots on a screen; each person receives feedback on both people's performances and payment received
Krueger	2007	Economic exchange	Trust game: An Investor invests money with a Trustee, and the Trustee decides to reciprocate or defect for a larger payoff
Saito	2010	Joint attention	Joint attention task: Each person shifts their gaze to a target object, cued either by the color of the ball or eye gaze of another participant
Krill	2012	Cooperation	Maze task: People either navigate through a maze with the help of an instructor or instruct a driver through the maze
Tanabe	2012	Joint attention	See Saito et al. (2010)
Stolk	2014	Communication	Communication task: Each person in a dyad is assigned a token; the Communicator is shown the goal configuration of both tokens and communicates this configuration using only their token; the Addressee infers the target placement of their own token
Spiegelhalder	2014	Communication	Autobiographical task: Each person shares or listens to autobiographical events
Bilek	2015	Joint attention	Joint attention task: Dyads aim to press the same target button; the Sender is shown the target location and communicates the target position with their eye gaze; the Receiver infers the target location
Koike	2016	Joint attention	Mutual gaze task: Dyads gaze at each other's face in real‐time and imagine what the other person is thinking; Joint attention task: one person follows their partner's eye movements, which are initiated either spontaneously or are cued
Shaw	2018	Economic games	Ultimatum game: A Proposer divides money between themselves and the Responder, and a Responder accepts or rejects the proposal
Abe	2019	Joint action	Force action task: Dyads work together to match the force of their grips to a target force
Koike	2019	Automatic mimicry	Gaze task: Participants either gaze into their partners' eyes and think of their partner or watch a video of their partner's eyes with a delay of 20 s
Špiláková	2019	Cooperation and competition	Pattern game: A Builder recreates a target pattern, and their partner acts as a Helper, Hinderer, or Observer
Goelman	2019	Joint attention	See Bilek et al. (2015)
Xie	2020	Collaboration	Pictionary: Three people take turns drawing a given word, evaluating others' drawings, and redrawing the word collaboratively in real‐time
Ellingsen	2020	Pain	Pain task: Patients receive moderately painful pressure to their leg and Clinicians apply either real, sham, or no treatment
Yoshioka	2021	Joint attention	Joint attention task: People either verbally identify a target object to initiate joint attention or respond to the initiator
Wang	2022	Cooperation and competition	“Cheap talk” game: A Receiver decides whether to take the Sender's suggestion of which box to open to win money

## 5 EMPATHY AS AN EXAMPLE

Hyperscanning using fMRI enables researchers to examine naturalistic, dynamic, group‐level neural activity. Here we bring together these strengths in an extended example of how fMRI hyperscanning can advance our understanding of social interaction:

Empathy is the ability to share in the affective or cognitive states of others, shaping how people respond to the needs of others and predict others' behaviors (Decety & Jackson, 2004). How are empathic processes elicited, and how do they impact others' thoughts and behaviors? With fMRI hyperscanning, one person (the discloser) could be asked to share a painful experience in their life. The other person (the respondent) would be free to engage in different behaviors: they may just listen, interject with backchanneling (e.g., saying “hmm”, “OK”), express empathy (“ouch!”), ask questions, share their own experiences, or interrupt and change topics altogether. When both the initial discloser and respondent are examined simultaneously, researchers have a unique opportunity to track the content and dynamics of these back‐and‐forths, and their consequences for the dyad.

For example, even with just these correlational data, researchers can address several questions: (1) *Natural real‐time responses*: Can naturally occurring behavioral responses be linked to specific types of neural responses? The respondent will be engaging in dynamic real‐time empathy, likely with large fluctuations in the extent to which they successfully make the discloser feel better. By pinpointing time points during the conversation during which the respondent is successfully empathic, we can work backward to see which real‐time empathic neural responses in the respondent predict success, and which neural responses in the discloser reflect their relief. (2) *Aligned responses*: Can dynamics between the two individuals predict the respondent's behaviors after engaging in empathic processes? Hyperscanning can uniquely identify moments of aligned activity across a dyad as one unique measure of successful empathy. We could then work backward to identify which empathic language most effectively elicit this alignment. (3) *Interdependent responses*: Can a respondent's behavior be predicted from just the brain responses of their own mind, or does it also depend on the discloser's brain and behavior? We can detect whether the discloser's experience of pain decreases as the respondent converses with the discloser, and how that change, in turn, impacts the respondents' own brain responses. Analyses that capture temporal dynamics between people, such as dynamic causal modeling (Marreiros et al., 2008) or Granger causality (Granger, 1969; Seth et al., 2015), can examine how responses in one brain predict brain responses in their partner's brain. For instance, we can examine how the respondent's activity in regions implicated in empathy influences the discloser's activity in limbic regions.

Prior work using a more conventional single‐subject approach laid the foundation for allowing us to know which neural signatures of empathy to look for (Shamay‐Tsoory, 2011; Zaki et al., 2009) At the same time, they could not have uncovered the types of insights that are unique to hyperscanning designs.

## 6 PRACTICAL CONSIDERATIONS FOR DOING fMRI HYPERSCANNING

Hyperscanning holds a great deal of promise. However, fMRI hyperscanning also comes with some real limitations, including hard limits to the paradigms that researchers can implement, and surmountable limits that researchers must confront in adopting a newer, complex methodology. Here are five considerations to address, both as individuals and as a community of researchers pursuing fMRI hyperscanning:

First, there are high‐level interpretation issues that hyperscanning researchers have yet to resolve. For instance, prior work links neural synchrony with mental synchrony and suggests a strong link between synchrony and social success (Wheatley et al., 2012). However, it is unclear what exactly neural alignment reflects (Hamilton, 2021; Holroyd, 2022), what features of a conversation or social interaction bring people into or out of alignment, and how these features support interaction success. These questions reflect a crucial gap in our understanding of how communicators synchronize and connect. On the one hand, these gaps provide opportunities to pursue fruitful research projects. On the other hand, these gaps may limit the interpretability of results. Because this area of research is in its infancy, there are myriad explanations for a given phenomenon (e.g., neural synchrony); stronger study designs will pit different explanatory variables against one another. For instance, if one hypothesizes that neural synchrony during a conversation tracks specifically with conversation enjoyment, they may want to also consider assessing related explanatory factors such as conversation comprehension or liking of the conversation partner.

Second, there are low‐level analytic issues to resolve. For instance, in fMRI, motion can negatively affect data quality: motion artifacts produce systematic decreases in and variable disruptions of fMRI signal (Power et al., 2012). People need to move their mouths when speaking, and state‐of‐the‐art methods of reducing speaking‐related motion (via personalized 3D printed head cases) do not appear to be effective (Jolly et al., 2020). To what extent might movement disrupt the quality of different neural measures (e.g., response magnitude in brain regions across the brain, spatial patterns, neural coupling)? Correlation‐based measures like ISC are likely to be impacted. That is, if people talk to each other in the scanner, their motions will look different from one another, potentially depressing otherwise strong ISC values. In a conversation study, spurious motion‐related activity may be time‐locked to speaking and listening turns, but not because of any interesting cognitive process happening during those turns. While any result would be less likely to reflect false positives, motion could hinder our ability to detect real and psychologically meaningful effects.

**FIGURE 4:**
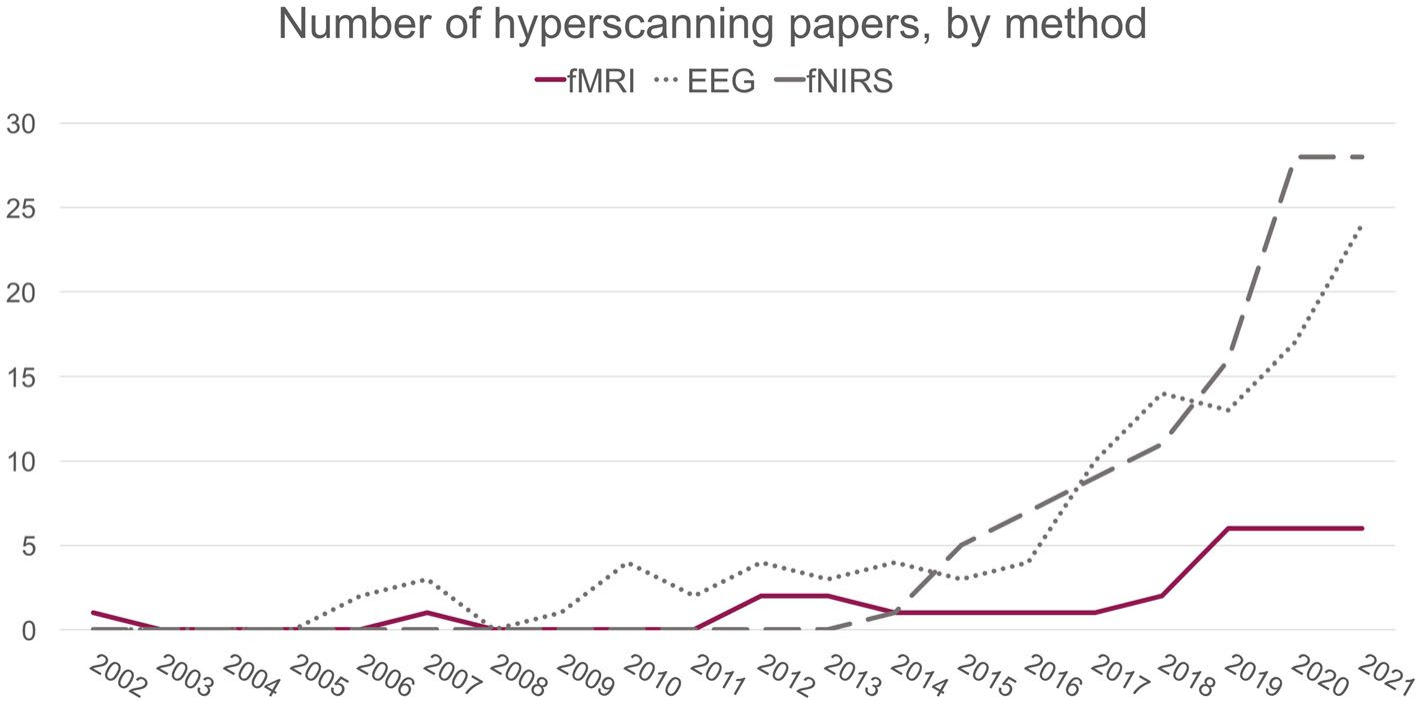
Counts of hyperscanning papers by method. Counts included all publications and proceedings indexed by dimensions.ai that contained the terms ‘hyperscanning’, the method (i.e., ‘fMRI’, ‘electroencephalogram [EEG]’, ‘functional near‐infrared spectroscopy [fNIRS]’), and excluded mentions of the other two methods in the title and/or abstract of the paper. We note that these counts may be imprecise as they include non‐empirical papers such as review papers and exclude papers that either mention more than one method or papers that do not use the term ‘hyperscanning’.

Third, since fMRI hyperscanning is relatively new (Figure 4), there is little standardization regarding norms for preprocessing or data analyses. Currently, popular analyses with hyperscanning include different variations of inter‐subject correlation (for a primer, Nastase et al., 2019), brain coherence (Cui et al., 2012), and inter‐subject functional connectivity (Simony et al., 2016). These measures all tap into the notion that brain synchrony, or lagged coupling, is informative. Some analyses, such as Granger causality analyses, aim to reveal temporal relationships (Granger, 1969; Seth et al., 2015). And with any newer analytical technique, fMRI hyperscanning offers us the opportunity to develop new tools to capture different dynamics, especially asymmetric dynamics (Wheatley et al., 2019). With so many analytic choices, it is important to have a clear idea of what you hope to get out of the data that can guide your analytic choices.

Fourth, one glaring pitfall is that the fMRI scanner does not provide a wholly natural context for interactions. People do not typically interact with others while lying down in a narrow tube, nor do interactions typically require people to maintain a very still position. Thus, hyperscanning with fMRI may not lend itself to fully naturalistic interactions. That said, interactions in the scanner can still be highly naturalistic: speaking in the scanner is akin to speaking to someone on the phone while being in a busy, noisy environment. Indeed, recent work has revealed the power voice has on social bonds: interactions including voice (phone, video chat, and voice chat) created stronger social bonds than interactions without (Kumar & Epley, 2021).

Finally, researchers may assume that for fMRI hyperscanning, they need to have two MRI scanners in the same MRI facility. However, as long as there is a good internet connection between the two stimulus computers, you can perform hyperscanning. With that said, logistics (mainly scheduling) may get trickier: you can only book when both scanners are available, which can raise issues if many researchers at each facility are using the scanner for their own projects.

## 7 FUTURE DIRECTIONS

Following Kurt Lewin's view that “Nothing is as practical as a good theory” (Lewin, 1943), we view the potential of hyperscanning in terms of applications to pressing problems in society. In particular, hyperscanning stands to provide new insight into how people connect, influence one another, and make sense of other minds.

Social connection is fundamental to human health and well‐being, and as such, serves as a practical end in itself. Successful social interactions engender social bonds that reduce stress, loneliness, and depression, and that support longevity (Eisenberger & Cole, 2012; Holt‐Lunstad et al., 2010). The link between social interaction and well‐being has been found not just for social interactions between close and intimate partners but also for shallower interactions between acquaintances (Holt‐Lunstad, 2018; Sandstrom & Dunn, 2014) and even complete strangers (Van Lange & Columbus, 2021). These findings reveal the general importance of social interactions for human health and happiness. Hyperscanning offers a way to understand these interactions by capturing naturalistic, real‐time, dyadic, and dynamic communication. Characterizing how interaction features and their neural substrates facilitate social connection is a natural next step.

Health is multiply determined by a range of social factors: Health behaviors spread through social networks and are influenced by norms (Smith & Christakis, 2008); successful doctor‐patient communication hinges on patients being able to successfully communicate what ails them and doctors being able to communicate diagnoses and influence patients (Heritage & Maynard, 2011; Ong et al., 1995); and racism and other forms of bias add harmful stress to individuals from marginalized groups (Williams et al., 1997), to name a few examples. Hyperscanning captures feedback loops created by people communicating with one another, enabling us to examine aspects of these loops that result in successful acts of information transmission and influence. For example, how does a patient's ability to ask questions and receive feedback influence their reception of doctors' messages? The stakes for communication and influence are high in health contexts, since they could lead to matters of life and death. And zooming out to consider planetary health, we can also examine, for instance, how one person's strategy to convince another to take action to protect the climate results in convergence or divergence of their brain responses, and, in turn, their thoughts, feelings, and actions.

The health of democracy, likewise, hinges on our ability to successfully discuss, debate, and ultimately create policy solutions that result from people's ability to get on the same page (Johnson & Johnson, 2000). On the other hand, communication failures are behind a range of intergroup struggles (Bruneau & Saxe, 2012). Hyperscanning can capture how people's mental representations converge after their discussions and efforts to reach a joint resolution.

Hyperscanning research is in its infancy. Researchers are beginning to use hyperscanning to better understand different social interactions (e.g., joint attention, conversation, cooperation). This area of research is an exciting field that has the potential to make positive contributions to our societies' most pressing concerns.

## CONFLICT OF INTEREST

All authors declare that they have no conflicts of interest.
